# Cross-species incompatibilities offer new insights into the functional consequences of satellite DNA evolution

**DOI:** 10.1007/s10577-026-09816-3

**Published:** 2026-09-26

**Authors:** Amber M. Ridgway, Piero Lamelza, Michael A. Lampson, Mia T. Levine

**Affiliations:** 1 Department of Biology, University of Pennsylvania, Philadelphia, PA USA; 2 Epigenetics Institute, University of Pennsylvania, Philadelphia, PA USA

**Keywords:** Satellite DNA, Rapid evolution, Genetic incompatibility, Chromosome segregation, DNA repair, Heterochromatin

## Abstract

Tandemly repeated, non-coding satellite DNAs are among the most rapidly evolving genetic elements in eukaryotic genomes, changing in identity, copy number, and genomic distribution. A growing list of empirical studies suggests that this rapid evolution has profound consequences on core biological processes. Here, we review recent studies that investigate the satellite DNA-mediated biology revealed by generating either inter-species hybrids or genotypes expressing divergent, satellite DNA-interacting proteins from a closely related species. The genetic incompatibilities that arise implicate chromosome segregation, DNA repair, the establishment of silent heterochromatin, and the spatial organization of chromosomes as essential processes vulnerable to the rapid evolution of satellite DNA. We discuss emergent themes from this relatively young body of literature and future challenges for the field.

## Introduction

Satellite DNAs are among the most rapidly evolving genetic elements in eukaryotic genomes, changing in identity, abundance, and genomic distribution (Jagannathan, et al. 2017; Cechova, et al. 2019; Logsdon and Eichler 2022; Naish and Henderson 2024). Replication slippage, unequal crossing over, and other recombination-based mechanisms contribute to this expansion, contraction, and homogenization (Smith 1976; Lower, et al. 2018; Thakur, et al. 2021). Species-specific copy number expansions undergo concerted evolution, which homogenizes repeat units within an array and drives even more extreme divergence between species (Garrido-Ramos 2017; Camacho, et al. 2022; Garrido-Ramos, et al. 2025). For decades, the extreme evolutionary plasticity of satellite DNA was attributed to a lack of functional constraint; that is, random mutation accumulation with no functional consequence. This observation, combined with the absence of any recognizable functional elements in satellite-rich DNA, led to minimal interest from the scientific community. This scientific neglect persisted for decades despite growing recognition that satellite DNA comprises a sizable fraction of eukaryotic genomes: about 10% of the human genome and more than 50% of others (Wheeler, et al. 1978; Garrido-Ramos 2017; McNulty and Sullivan 2018; Mora, et al. 2023).

This scientific neglect was reinforced by multiple technical barriers to simply describing satellite DNA sequence and organization. First, the instability of cloned DNA repeats in bacteria was a major barrier to early sequencing technology, such as Sanger sequencing. Second, even when Sanger sequencing successfully traversed a repeat array, assembly algorithms could not assign the relatively short, non-unique sequence reads to a single genomic location. This second challenge plagued next-generation whole-genome sequencing technology, which produced only short reads. These short reads proved powerful for genome-wide cataloging of individual satellite DNA sequences and for guiding cytogenetic investigation of satellite DNA organization (Novak, et al. 2013, 2017; Ruiz-Ruano, et al. 2016; Lower, et al. 2018). However, this unassembled satellite DNA left large gaps in eukaryotic genome assemblies long after genome sequences were declared “complete” (Lander, et al. 2001; Venter, et al. 2001). This included the 2003 announcement of the complete human genome, which, in fact, was missing 10% of the sequence. In 2003, this omission was of minimal concern given the negligible evidence of satellite DNA functionality.

Over the past two decades, an accumulating body of evidence rejects the notion that satellite DNA evolution is functionally inconsequential. This literature, primarily from *Drosophila* and mice, instead demonstrates the profound impacts on core biological processes and even the emergence of reproductive barriers between species driven by satellite DNA evolution (see below). These insights have not arisen from classical genetic approaches, i.e., mutating or deleting a genetic element and then assaying for a functional consequence. Indeed, manipulation of the vast stretches of satellite DNA remains a challenge, though some progress has been made (Eickbush, et al. 2026; Gilmour, et al. 2026; Wang, et al. 2026). Instead, major progress in detecting the functional consequences of satellite DNA evolution comes from studies that leverage its rapid sequence evolution as a “natural manipulation”.

**Fig. 1:**
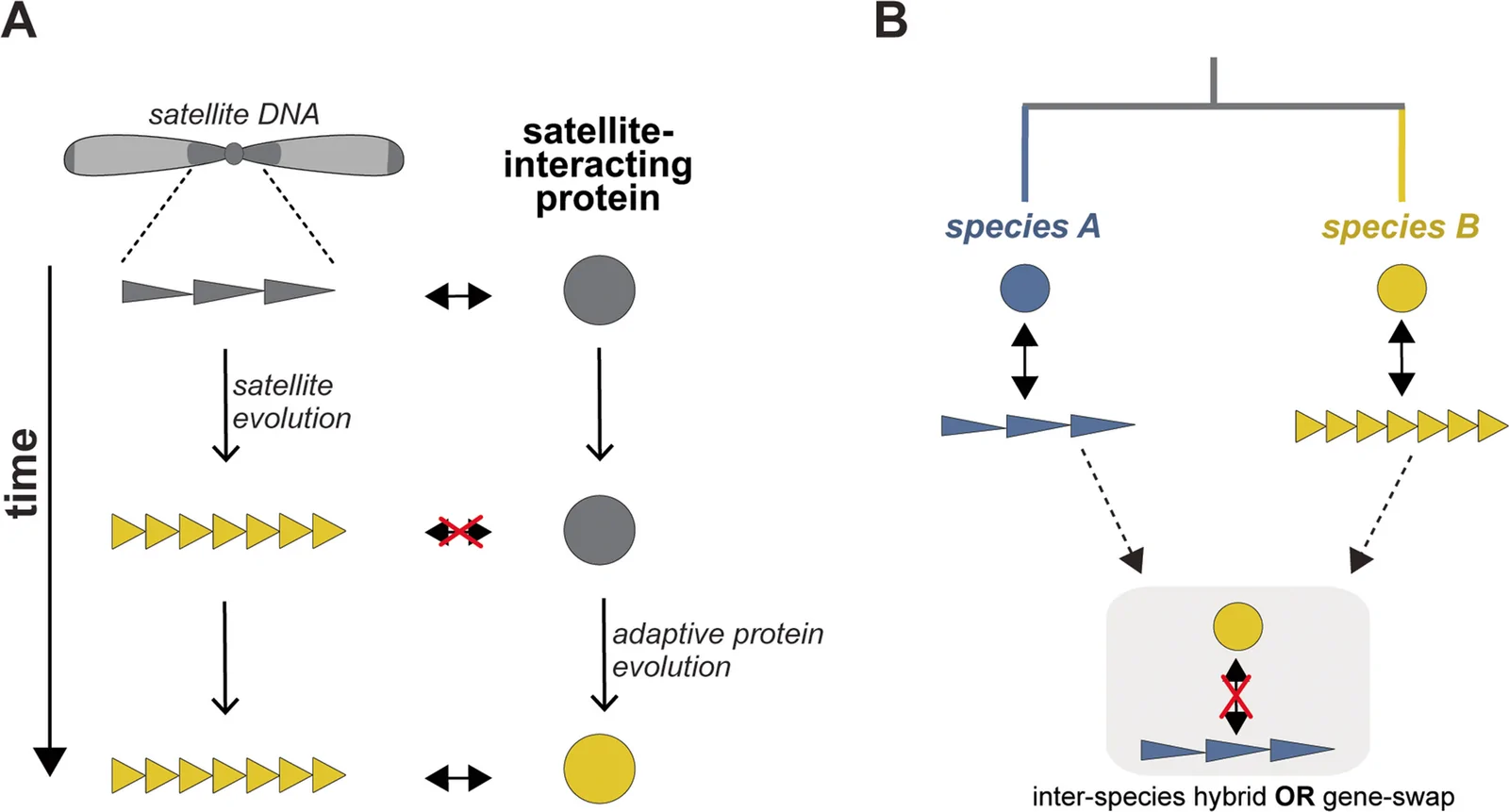
Model of coevolution between a satellite DNA and a satellite DNA-interacting protein and the logic of experimental manipulations. **A** Model of antagonistic coevolution between satellite DNA and a satellite DNA-interacting protein over evolutionary time. Satellite DNA, concentrated around telomeres and centromeres, evolves rapidly. Evolution of a novel sequence and/or change in copy number reduces the host’s fitness, selecting for adaptive non-synonymous changes at a satellite-interacting protein. Under a coevolutionary model, species-specific proteins are required to block satellite proliferation or to mitigate the collateral damage of proliferation. **B** Species A and Species B have distinct complements of satellite DNA and encode species-specific satellite-interacting proteins. The biology sculpted by this coevolution can be studied by generating either interspecific hybrids or transgenic organisms that encode the divergent, species-specific proteins from a closely related species. The red ‘X’ indicates an incompatibility between a satellite array from one species and a satellite-interacting protein of another. The DNA–protein interaction may be direct, but more often, it is indirect

The radical differences in satellite DNA sequence, copy number, and organization between even closely related species have enabled cross-species experiments that reveal satellite DNA function. These studies are grounded in the conceptual framework of species-specific coevolution between satellite DNA and satellite DNA-interacting proteins (Fig. 1A). Under this model, rapid sequence changes of satellite DNA spur adaptive changes in proteins to maintain their binding, packaging, processing, or repair of this ever-changing substrate. A history of coevolution results in compatible, species-specific versions of proteins and satellite DNA (Henikoff, et al. 2001; Brand and Levine 2021). When a rapidly diverged, species-specific DNA satellite encounters a different species’ version of a satellite-interacting protein, fertility and/or viability are compromised (Fig. 1A). Studies that probe the molecular and cell biological basis of this reduction in fitness have revealed the biological pathways that require coevolution between these two genome components and, importantly, evidence of functionally consequential satellite DNA evolution.

Two distinct genetic approaches have been utilized to force an encounter between incompatible satellite DNA and satellite DNA-interacting proteins: crossing closely related species to create hybrid genotypes, or conducting inter-species swaps of fast-evolving, satellite DNA-interacting proteins (Fig. 1B). Closely related but reproductively isolated species share high sequence identity across most of their genome, indicating that the two genomes are largely “compatible” in the hybrid. Only functionally diverged sequences have the potential to trigger the genetic incompatibilities that lead to or reinforce reproductive barriers. If satellite DNA evolution has spurred protein adaptation along one or both species’ lineages, the inter-species hybrid may exhibit dysfunction. By closely studying the impaired function in hybrids, we can learn about the biology that depends on, or persists in the face of, satellite DNA evolution. Evidence of functionally consequential satellite DNA evolution can also be revealed by identifying and manipulating the proteins adapted to specific satellite DNA sequences or satellite-rich genomic regions. Replacing an endogenous satellite-interacting protein with one from a close relative will imperil the satellite DNA-regulated biology that depends on the species-specific version.

Here, we review these studies to highlight the empirically demonstrated, major cellular processes sculpted by satellite DNA evolution. These processes include chromosome segregation, the establishment of silent chromatin packaging, DNA repair, and the preservation of chromosome structure and 3D genome organization. Reviewing these studies reveals that this subfield has matured from one seeking to establish *whether* satellite DNA evolution has functional consequences to now asking *how* these dynamic sequences perturb and preserve core biological processes.

## The impact of satellite DNA evolution on chromosome segregation

### The centromere and kinetochore

Centromeres are essential chromosomal loci that recruit the proteins required to assemble kinetochores, the large proteinaceous complexes that interact with spindle microtubules to segregate chromosomes during cell division. Despite their conserved role in chromosome segregation across eukaryotes, centromeres are typically built on rapidly evolving satellite DNA or other repetitive sequences (Melters, et al. 2013; Chang, et al. 2019; Balzano and Giunta 2020; Thakur, et al. 2021; Logsdon and Eichler 2022; Naish and Henderson 2024). This paradoxical observation is partially resolved by epigenetic specification of centromeres in most eukaryotes by the centromere-specific histone H3 paralog, CENP-A. CENP-A-containing nucleosomes recruit all other centromeric proteins, even in the absence of satellite DNA, and therefore are the defining epigenetic mark of centromeres (Depinet, et al. 1997; Henikoff, et al. 2001; Black, et al. 2007; Piras, et al. 2010; Bassett, et al. 2012; Hori, et al. 2013; Jaggi, et al. 2026). Many of these essential centromere proteins, including CENP-A, evolve under positive selection across the eukaryotic tree (Malik and Henikoff 2001; Talbert, et al. 2002, 2004; Schueler, et al. 2010; Finseth, et al. 2015; Kumon, et al. 2021). This evolutionary signature is usually observed in immune genes that recurrently adapt to rapidly evolving pathogens, suggesting that centromere proteins continuously adapt to ever-changing satellite DNA (Fig. 1A). This coevolutionary process, in addition to epigenetic specification, offers a resolution to the “centromere paradox”.

To explain the agent of selection spurring this coevolution, the centromere drive hypothesis was formulated (Henikoff, et al. 2001). This hypothesis proposes that centromeric satellite DNAs act as selfish genetic elements that select for centromere protein innovations, which suppress their selfish activity. In many organisms, female meiosis is asymmetric, with homologous chromosomes randomly segregating during meiosis I either towards the single future egg cell or degenerative germ cells (“polar bodies”), with either outcome equally likely. However, selfish centromere DNA variants bias their inheritance to the next generation by preferentially attaching to microtubules emanating from the spindle pole oriented towards the future egg cell. Fitness costs of these selfish centromeres, such as nondisjunction, select for novel centromere protein variants (Henikoff, et al. 2001).

Several lines of evidence support the centromere drive hypothesis, which we briefly summarize here because more comprehensive reviews on this topic have been published recently (Clark and Akera 2021; Dudka and Lampson 2022; Kumon and Lampson 2022; Talbert and Henikoff 2022; Finseth 2023). The clearest genetic evidence of centromere drive comes from inter-species hybrids of the yellow monkeyflower, in which the centromere-associated D-locus is transmitted to 98% of offspring during female meiosis (Fishman and Willis 2005). The D-locus carries an expansion of the centromeric Cent728 satellite, strongly implicating a direct role for centromeric DNA (Fishman and Saunders 2008).

Molecular mechanisms underlying preferential transmission in mammals are being uncovered using mouse hybrids whose parental strains differ in the amount of their centromeric “minor satellite”. Centromeres with very small minor satellite arrays have less CENP-A chromatin, build smaller kinetochores, and preferentially orient toward the polar body when paired with larger minor satellite arrays in meiosis I (Chmatal, et al. 2014; Iwata-Otsubo, et al. 2017). The biased orientation depends on an inherent asymmetry of the oocyte spindle, with larger centromeres more readily detaching from tyrosinated microtubules enriched at the cortical side of the spindle and reorienting toward the egg (Akera, et al. 2017). This reorientation depends on microtubule-destabilizing proteins recruited through the kinetochore, which is larger on larger centromeres (Akera, et al. 2019).

The centromere drive hypothesis predicts that driving centromeres impose a fitness cost (Malik and Henikoff 2001, 2009; Pan and Akera 2025). This prediction is supported by monkey flowers, where plants homozygous for the D-locus produce fewer seeds and less pollen (Fishman and Saunders 2008; Fishman and Kelly 2015), although the underlying mechanisms remain unclear. Given this cost, centromere proteins are predicted to adapt in ways that suppress drive or its consequences. Indeed, an unlinked suppressor of D-locus drive maps to a region of the genome containing the centromeric histone CenH3, which also exhibits signatures of a selective sweep around the same time that the D-locus arose (Finseth, et al. 2015, 2021). These data are consistent with CenH3 adaptation in response to the driving centromere; however, finer mapping or transgenic experiments are required to link the two definitively.

**Fig. 2:**
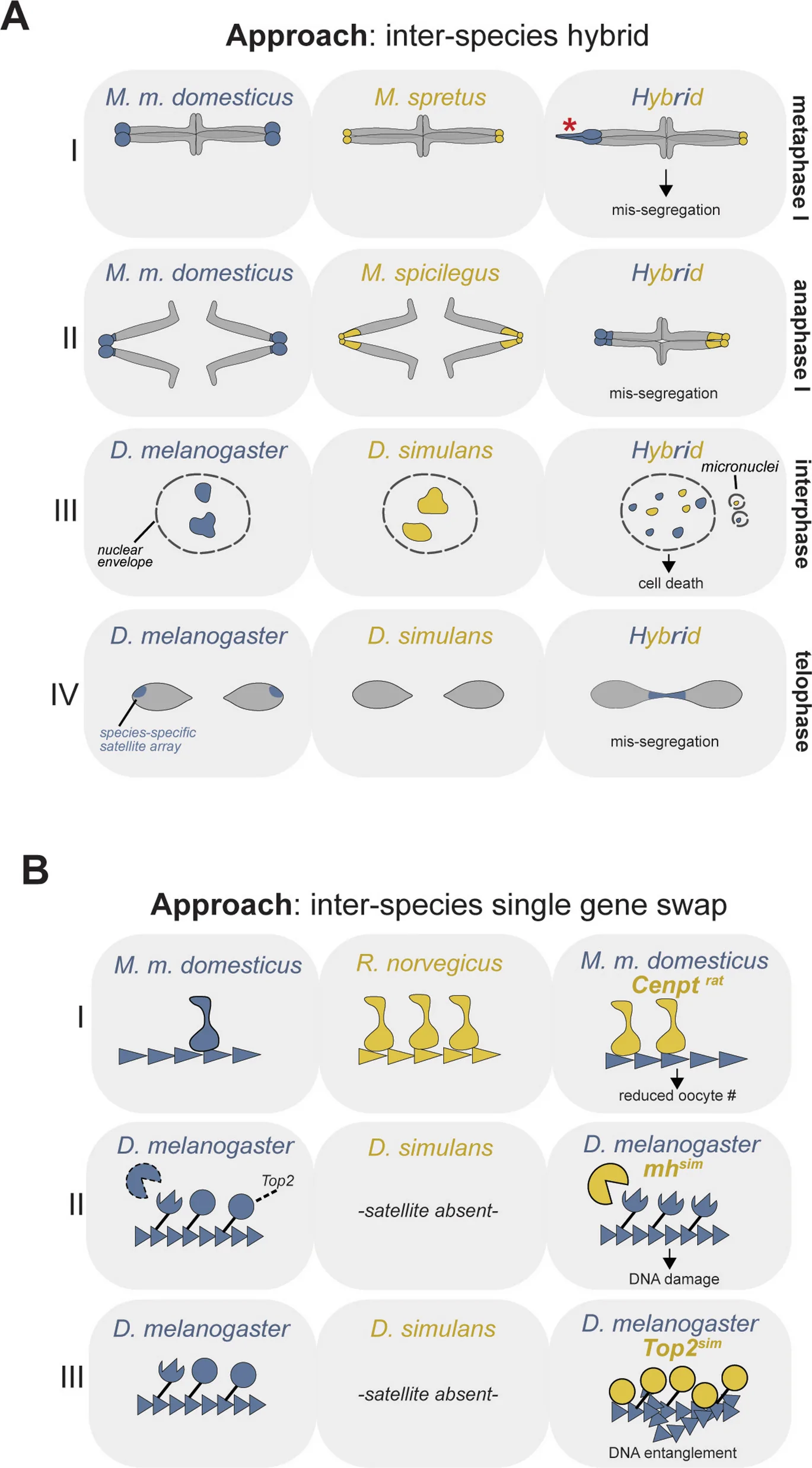
Schematics of the consequences of experimentally disrupting coevolution between satellite DNAs and satellite DNA-interacting proteins using two approaches. **A** Generating inter-species hybrids results in (I) centromere stretching at metaphase I during female meiosis, linked to reduced condensin (El Yakoubi and Akera 2023), (II) failure of cohesin degradation at anaphase I of female meiosis, leading to aneuploidy (El Yakoubi, et al. 2026), (III) disassociation of chromocenters, leading to micronuclei formation (Jagannathan and Yamashita 2021), and (IV) mis-segregation of a species-specific satellite in the early embryo (Ferree and Barbash 2009). **B** Swapping or expressing a single, adaptively evolving gene from one species into another results in (I) hyperaccumulation of a centromere-binding protein (though the binding is sequence-independent (Dudka et al. 2025b)), (II) satellite-linked DNA damage caused by premature degradation of essential DNA–protein crosslinks (Brand and Levine 2022), and (III) satellite DNA entanglements that cause chromosome mis-segregation (Brand, et al. 2026)

If centromere proteins adapt to counteract the cost of selfish satellites, then reversing that adaptation should reveal the cost, as demonstrated for mouse CENP-T (Dudka et al. 2025b). CENP-T’s histone fold domain can directly bind DNA (Nishino, et al. 2012; Pesenti, et al. 2022; Yatskevich, et al. 2022) and is evolving under positive selection in rodents (Kumon, et al. 2021), suggesting that it may be directly adapting to selfish satellite DNA. This functional innovation was experimentally “reversed” by overexpressing CENP-T variants in which the mouse histone fold domain was replaced with that of closely related rodents or with a reconstructed ancestral domain (Dudka et al. 2025b). The chimeras encoding orthologous domains increased CENP-T binding to centromeres in mouse oocytes (Fig. 2B.I), indicating that the mouse protein evolved to reduce binding to its own centromeres. Furthermore, female transgenic mice with the rat histone-fold domain swapped into the endogenous locus had a reduced number of oocytes, demonstrating an organismal cost when CENP-T adaptation was experimentally reversed.

The authors inferred that CENP-T evolved adaptively to reduce kinetochore size after a driving centromere was fixed, rather than to block an actively driving centromere. Analyses of CENP-T binding to distinct satellite arrays in mouse or to divergent satellites in other species suggested that innovation in centromere binding of CENP-T is independent of satellite DNA sequence. Consequently, evolution to reduce binding across homologs with asymmetric satellites (where one centromere is driving) would reduce kinetochore size on both homologs, conferring no advantage to a more weakly-recruited CENP-T variant. However, if a selfish variant that recruits more centromere proteins fixes in the population, CENP-T evolution can reduce kinetochore size and restore normal kinetochore-microtubule attachments on all chromosomes, thereby mitigating the fitness cost imposed by the selfish centromere.

Together, these systems establish the centromere drive hypothesis as a useful framework for understanding centromere evolution and its impact on chromosome segregation, although several aspects remain unresolved. Mouse studies suggest that very small satellite arrays can limit the amount of centromere chromatin, as discussed above, but the underlying mechanisms are unclear. Once arrays are larger than this minimal size, the impacts of further expansions are also unclear, as centromere chromatin does not scale with array size (Ma, et al. 2026). The mechanisms by which other satellite variants may bias their transmission have not yet been investigated, and the organismal consequences of the resulting protein adaptations have been measured in only a few cases. Moreover, directly testing whether protein changes are direct consequences of satellite changes remains a major challenge.

### The condensin complex

Faithful chromosome segregation requires genome-wide chromatin compaction by the condensin complexes, which act during mitosis and are repurposed during meiosis to support the specialized segregation of homologs and sister chromatids (Freeman, et al. 2000; Bhalla, et al. 2002; Chan, et al. 2004; Viera, et al. 2007). Condensin-mediated chromatin compaction is achieved through DNA loop extrusion (Hirano 2005; Ganji, et al. 2018). This activity is important genome-wide; however, compaction is especially critical at satellite-rich centromeres and pericentromeres where chromosomes must withstand spindle forces (Hagstrom, et al. 2002; Samoshkin, et al. 2009). Indeed, depletion of condensin II not only disrupts compaction along chromosome arms but also causes pericentric satellite DNA to stretch, leading to chromosome mis-segregation (Oliveira, et al. 2005; Nishide and Hirano 2014). The rapid evolution of pericentric satellite DNA raises the possibility that these genomic loci may have species-specific requirements for efficient loop extrusion by the condensin complex. Such species-specific requirements would select for evolutionary innovation in this vital complex.

Consistent with chronic pressure on condensin complexes to innovate, positive selection shapes multiple subunits of condensin II across insects and multiple mammalian lineages (Beck and Llopart 2015; King, et al. 2019; Forni, et al. 2024). These subunits include both those that contact DNA and those that do not. Notably, these signatures are detectable even between closely related sister species of *Drosophila* (Beck and Llopart 2015), implicating a recent and recurrent evolutionary pressure. Whether recent and recurrent pericentric satellite evolution is the source of this evolutionary pressure remains untested. However, new data implicate satellite DNA evolution in selecting for innovation in condensin *regulation*.

This recent study exploited mouse hybrids to investigate how pericentric satellite evolution may shape chromatin compaction. The “major satellite” arrays that comprise the model *Mus musculus domesticus* pericentromeres span multiple megabases, whereas those of *M. spretus* are present but barely detectable (Wong, et al. 1990; Galtier, et al. 2004; Suzuki, et al. 2004). Crossing these two species generates first-generation hybrid daughters that are subfertile. The oocytes of these subfertile hybrid females display meiotic chromosome mis-segregation and aneuploidy. Intriguingly, chromosomal condensin II levels are markedly lower in *M. spretus* oocytes than in *M. musculus* oocytes, and hybrid oocytes recapitulate the *M. spretus*-like, reduced levels. Consequently, the massive arrays of *M. musculus* major satellite in the hybrid encounter an unfamiliar, low dose of condensin II. At meiotic prometaphase I, the hybrid females exhibit striking decompaction and stretching of the *M. musculus* expanded pericentromeres (Fig. 2A.I). This defect can be rescued by overexpression of the condensin II subunit, Cap-G2 (El Yakoubi and Akera 2023). These findings indicate that in hybrid oocytes, species-specific expanded pericentric satellites lack sufficient condensin levels for DNA compaction.

What is the mechanism of condensin reduction at the major satellite in these mouse hybrids? Staining in hybrid oocytes for various pericentromeric proteins revealed aberrantly high levels of Topoisomerase 2A (TOP2A) at the decondensed major satellite arrays. This over-recruitment of TOP2A is sufficient to reduce condensin and induce DNA stretching: ectopic targeting of TOP2A to smaller *M. spretus* pericentromeres recapitulated both the aberrant condensin signal and aberrant DNA topology. Together, these results suggest that elevated TOP2A may alter major satellite topology (for example, by aberrantly reducing DNA supercoiling) required for condensin recruitment and/or retention, ultimately leading to failed chromosome segregation. Why TOP2A is over-recruited to the major satellite is not yet clear. However, intriguingly, Top2A evolves adaptively in rodents, raising the possibility of coevolution between Top2A and the major satellite, with consequences for condensin recruitment and ultimately, chromosome transmission. Moreover, the functional consequences of adaptive evolution of condensin itself also await investigation.

### The cohesin complex

During meiosis, faithful chromosome segregation depends, in part, on the cohesin complex. From S phase to anaphase I, cohesin spans the entire chromosome axis, ensuring sister chromatid cohesion, meiotic recombination, and proper kinetochore orientation on the meiotic spindle (Ochs and Gerlich 2026). At anaphase I, this multimeric complex is degraded along the chromosome arms but retained at centromeres and pericentromeres. At anaphase II, the remaining centromeric and pericentromeric cohesin is completely degraded, allowing sister chromatids to segregate into haploid gametes. This sister chromatid cohesion is essential for resisting spindle microtubule forces that pull chromatids toward the poles: cohesin depletion results in premature sister chromatid separation and aneuploidy. Premature cohesin breakdown, as observed in the oocytes of women of advanced maternal age, is a leading cause of age-related meiotic chromosome segregation errors and trisomy births (Chiang, et al. 2012; Herbert, et al. 2015).

Chromosome-wide localization of the cohesin complex, at least before meiotic (and mitotic) anaphase, suggests a lack of sequence-specificity. Indeed, cohesin protection from removal depends on the presence of a histone mark rather than a specific DNA sequence (Kawashima, et al. 2010). However, many cohesin subunits show strong signatures of positive selection across *Drosophila* and mammalian lineages (King, et al. 2019; Forni, et al. 2024). This includes the mammalian mitotic subunit, RAD21 and the meiotic paralogs, REC8 and RAD21L. In addition, the protein that protects meiotic cohesin from degradation – Shugoshin 2 (SGO2) – also evolves under positive selection (Kumon, et al. 2021; Pontremoli, et al. 2021). These data implicate a chronic evolutionary pressure on cohesin subunits to innovate. Despite these sequence footprints typical of coevolution, and cohesin enrichment at satellite-rich centromeres and pericentromeres during meiosis II, there have been no direct tests of the functional significance of cohesin evolution. Future empirical work, such as inter-specific gene swaps, may illuminate how and why cohesin subunits and other members of this pathway evolve adaptively.

While direct investigation of the functional consequences of cohesin adaptive evolution is lacking, defects found in an inter-species hybrid implicate rapid functional divergence of the cohesin pathway. In oocytes of the hybrid daughters of *M. musculus domesticus* and *Mus spicilegus*, homologous chromosomes fail to separate during meiosis I, leading to aneuploid eggs (El Yakoubi, et al. 2026). The authors discovered that, while a cohesin complex subunit appeared only at the centromeres and pericentromeres during meiosis II in the pure species,  this subunit decorated the length of the chromosome arms in the hybrid. Moreover, the cohesin complex subunit was not the only ectopically localized protein. SGO2, which is also normally confined to the centromeres where it protects pericentromeric cohesin from degradation, decorates the chromosome arms as well. Moreover, the histone mark that recruits SGO2 to pericentromeres (phosphorylated H2AT121) was also aberrantly distributed chromosome-wide, as was the chromatin writer of this mark, BUB1. Together, these findings suggest that, in these *Mus* inter-species hybrids oocytes, spatial restriction of cohesin protection to the major satellite-enriched pericentromeres is disrupted (Fig. 2A.II). The cause of this disruption has yet to be elucidated, but it is interesting that the centromeric minor satellite arrays of *Mus spicilegus* are significantly smaller than those of *M. musculus domesticus*, while their pericentromeres (‘major satellite’) are significantly larger (Ostromyshenskii, et al. 2011).

Intriguingly, crosses between two species from a different mouse genus, *Peromyscus,* similarly revealed aberrant sister chromatid separation. However, the chromosomal defect in these hybrids showed the opposite pattern. Rather than aberrant overprotection of cohesin from degradation in the hybrid, the authors observed the loss of cohesin. This loss of cohesin in hybrids was associated with lower levels of both BUB1 kinase and its phosphorylation of H2AT121, which recruits SGO2. These data, together with the *Mus* hybrids, further implicate the evolutionary lability of cohesin-mediated, faithful chromosome separation, if not the direct functional consequences of the evolution of cohesin itself. Indeed, the molecular evolution studies of cohesin implicate amino acid changes in directly altering cohesin function, while these two inter-species hybrids implicate the rapid evolution of cohesin regulation. Future research may define the relationship between these striking indicators of rapid protein evolution and the potential for satellite DNA divergence to drive it.

## The impact of satellite DNA evolution on the establishment of heterochromatin silencing

Despite the extremely rapid sequence turnover of satellite DNA (Melters, et al. 2013; Wei, et al. 2018; Arora, et al. 2021; Thakur, et al. 2021; Logsdon and Eichler 2022), packaging of this sequence into transcriptionally quiescent constitutive heterochromatin is conserved across plants and animals (Soppe, et al. 2002; Guenatri, et al. 2004; Krassovsky and Henikoff 2014; Jagannathan, et al. 2018; Xu, et al. 2026). Heterochromatin packaging is characteristic of the large tandemly repeated satellite arrays that are typically located at pericentromeres, but satellites also occur as short and dispersed arrays within euchromatin, whose chromatin state is more variable (Kuhn, et al. 2012; de Lima, et al. 2020). A hallmark of heterochromatin is the enrichment of nucleosomes marked by histone H3, lysine 9 di/tri methylation (H3K9me2/3). This epigenetic mark recruits heterochromatin protein 1 (HP1), which recruits a myriad of downstream effectors that transcriptionally silence and regulate replication (Lima-de-Faria and Jaworska 1968), repair (Chiolo, et al. 2011; Tsouroula, et al. 2016), and the nuclear position of the underlying satellite arrays (Janssen, et al. 2018). This packaging is essential for protecting genome integrity: disruption of heterochromatin is associated with DNA damage, chromosome rearrangements, and/or chromosome instability (Peters, et al. 2001; Peng and Karpen 2007, 2009). In addition to maintaining genome integrity of repeat-rich DNA, heterochromatin packaging also contributes to pericentromere function during mitosis through sister-chromatid cohesion (Bernard, et al. 2001; Nonaka, et al. 2002; Hahn, et al. 2013) and the recruitment of the Chromosome Passenger Complex (CPC) (Abe, et al. 2016; Sako, et al. 2024), a key regulator of kinetochore-microtubule attachments required for accurate chromosome segregation. Given the strict conservation of these heterochromatin functions, it appears paradoxical that such diverse, fast-evolving satellite sequences are so faithfully recognized and packaged into silent chromatin. Recent data are beginning to shed light on this evolutionary conundrum.

A classic, intuitive resolution of this conundrum is that the primary DNA sequence is inconsequential for heterochromatin packaging. Under this framework, heterochromatin propagates only through epigenetic mechanisms. Consistent with this, SUV39 methyltransferases, which catalyze H3K9me2/3, and HP1 both contain chromodomains that recognize H3K9me3 directly (Jacobs and Khorasanizadeh 2002; Wang, et al. 2012). SUV39 binding to existing H3K9me3 promotes deposition of the same mark on neighboring nucleosomes, while HP1 binding both recruits additional SUV39 and scaffolds the downstream effectors that establish the heterochromatin environment. This self-propagating epigenetic circuit can theoretically maintain heterochromatin against the diluting effects of DNA replication and cell division, independent of the underlying DNA sequence (Grewal 2023).

A major prediction of this purely epigenetic propagation model is that the proteins responsible for heterochromatin packaging are as conserved as heterochromatin packaging itself. However, genes involved in heterochromatin regulation are among the fastest-evolving in eukaryotic genomes. A systematic analysis of 106 heterochromatin-related genes in *Drosophila* found that a significantly higher fraction of these genes, including the SUV39 histone methyltransferase, show signatures of adaptive protein evolution than a random set of genes (Lin, et al. 2024). This systematic analysis extends previous reports focused on the rapid evolution of individual heterochromatin proteins, including HP1 paralogs (Vermaak, et al. 2005; Levine, et al. 2012; Helleu and Levine 2018), and ZAD-ZNFs (Kasinathan, et al. 2020). The prevalence of fast evolution is difficult to reconcile with a purely epigenetic model of heterochromatin maintenance. Instead, it suggests that rapid evolution of the underlying satellites packaged by these factors require recurrent heterochromatin innovation. This systematic signal of adaptive protein evolution provides indirect evidence that the underlying satellite sequence may dictate heterochromatin packaging.

Once established, heterochromatin packaging can be epigenetically propagated and so sequence-independent. However, sequence may dictate the establishment of heterochromatin in the early embryo, when chromatin structure forms de novo in the absence of pre-existing H3K9me3 epigenetic templates. In mice and flies, for example, near-complete replacement of histones by protamines during spermiogenesis means the paternal genome arrives at fertilization without H3K9me3 to template histone methyltransferases (Braun 2001; Loppin, et al. 2005; Puschendorf, et al. 2008). In species with rapid early cleavage divisions (including flies, fish, and frogs), short S/M cycles have been proposed to dilute heterochromatin modifications faster than they can be rewritten (Seller, et al. 2019; Fukushima, et al. 2024). In both scenarios, the self-propagating epigenetic circuit cannot contribute to heterochromatin, suggesting satellite DNA may specify where heterochromatin assembles. Therefore, the early embryo is a physiologically relevant context in which satellite sequence evolution may impact heterochromatin packaging. If heterochromatin proteins coevolve with fast-evolving satellites to maintain heterochromatin packaging, inter-species incompatibilities would manifest specifically in hybrid embryos. Here, maternally provisioned trans-acting heterochromatin proteins, adapted to maternal satellite sequences, may fail to recognize and package the divergent paternal satellites encountered at fertilization.

### Sequence-dependent PRC1 and PRC2 heterochromatin establishment in early mouse embryos

The best-characterized example of satellite DNA sequences directing de novo heterochromatin establishment is in the early mouse embryo. During the first four cell cycles, paternally inherited satellites lack H3K9me3 and are instead packaged by Polycomb Repressive Complexes 1 and 2 (PRC1 and PRC2) into heterochromatin marked by H2AK119ub1 (H2Aub) and H3K27me3 (Puschendorf, et al. 2008; Saksouk, et al. 2014; Tardat, et al. 2015). These marks are later replaced with H3K9me3, but their initial appearance represents a well-established example where satellite DNA directs de novo heterochromatin formation. The CBX2 subunit of PRC1 recognizes satellite DNA via its AT-hook (Tardat, et al. 2015), which binds to the narrow minor grooves of double-stranded DNA generated by stretches of tandem A/T nucleotides (A/T runs) (Kawaguchi, et al. 2017; Battista, et al. 2024). PRC2 localization likely requires BEND3, which binds *M. musculus* pericentric major satellite DNA in vitro (Saksouk, et al. 2014).

**Fig. 3:**
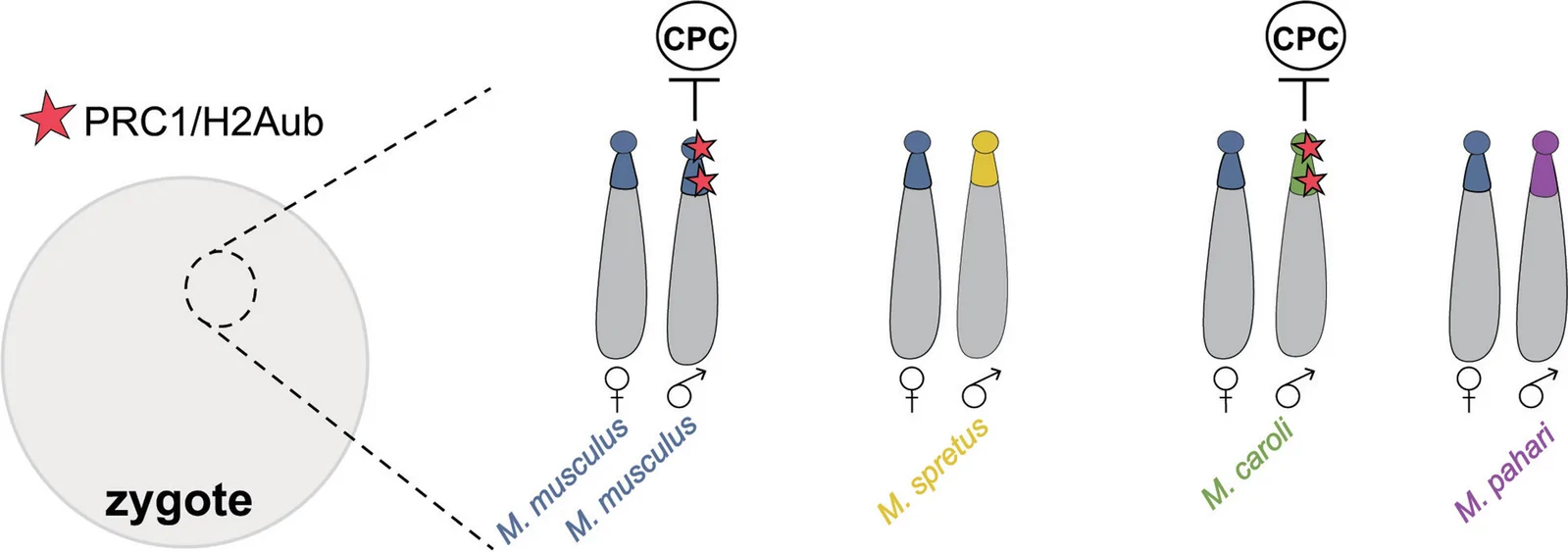
Satellite DNA sequence composition determines recognition and packaging of paternally inherited satellites into heterochromatin. In the hybrid zygotes with maternal *Mus musculus* chromosomes and paternal chromosomes from *M. musculus *or closely related species (*M. spretus, M. caroli* and *M. pahari*), only paternally inherited *M. musculus* and *M. caroli* satellites are recognized by Polycomb Repressive Complex 1 (PRC1) and packaged into H2AK119ub1 (“H2Aub”) heterochromatin. H2Aub inhibits the recruitment of the Chromosome Passenger Complex (CPC), a key regulator of kinetochore-microtubule attachments, and is associated with an increased frequency of lagging paternal chromosomes in anaphase

Lamelza et al. used closely related species of mice to test whether natural divergence in satellite sequence altered their recognition and packaging by either Polycomb complex (Lamelza, et al. 2026). By fertilizing *M. musculus* eggs with sperm from progressively divergent species (*M. spretus, M. caroli,* and *M. pahari*), they found that maternally provisioned *M. musculus* PRC1 robustly packaged *M. musculus* and *M. caroli* paternal satellites but not those of *M. spretus* and *M. pahari*. The satellites that recruited PRC1 were enriched for the specific A/T runs preferred by the CBX2 AT-hook, whereas the satellites that did not recruit PRC1 were depleted for this sequence motif. Together, these data indicate that paternal satellite sequence dictates PRC1 recruitment and heterochromatin assembly (Fig. 3).

Crucially, these sequence-based differences in heterochromatin assembly appear to impact pericentromere function during mitosis. Although PRC1 is essential for the integrity of paternal *M. musculus* major satellite arrays (Liu, et al. 2020), the H2AK119ub1 modification that it catalyzes inhibits the acquisition of an adjacent histone modification (H2AT121phos) required for the recruitment of the CPC (Lamelza, et al. 2026). The presence of H2AK119ub1 coincides with reduced CPC at paternal *M. musculus* pericentromeres compared to maternal *M. musculus* and paternal *M. pahari* pericentromeres, which are not enriched for H2AK119ub1 (Fig. 3). Consistent with CPC function in regulating kinetochore-microtubule interactions, reduced CPC is associated with more frequent segregation errors of paternal compared to maternal *M. musculus* chromosomes during the first embryonic mitosis. This study therefore highlights how satellite sequence evolution leads to species-specific differences in both heterochromatin assembly and pericentromere function in the early embryo.

Given that Polycomb complexes establish heterochromatin on rapidly changing satellites in the mouse embryo through direct sequence recognition, Polycomb subunits are predicted to recurrently adapt to species-specific satellites to maintain their heterochromatin packaging. However, the evidence of such adaptive evolution is limited. Molecular evolution analyses of Polycomb subunits have so far been restricted to *Drosophila* (Calvo-Martin, et al. 2016; Lin, et al. 2024), where some show the expected signatures of adaptive evolution, but the role of Polycomb in establishing heterochromatin in the *Drosophila* embryo has yet to be explored directly. In mice, molecular evolution analysis of Polycomb group proteins has not been conducted. Nevertheless, we note that vertebrates encode multiple PRC1 complexes that share the core RING1A/RING1B ubiquitin ligase but differ in their chromatin-targeting subunits (Geng and Gao 2020). It is possible that different chromatin-targeting subunits are deployed in different species to ensure heterochromatin establishment. This predicts that *M. spretus* and *M. pahari*, whose satellites are not recognized by the PRC1 complex in *M. musculus* eggs, establish heterochromatin packaging of their own satellites through an alternative targeting subunit in PRC1. This possibility remains to be tested.

How broadly Polycomb contributes to heterochromatin establishment at embryonic satellites also remains to be determined. Interestingly, H3K9me3 heterochromatin is retained at satellites during human spermiogenesis and therefore directly inherited by the zygote (van de Werken, et al. 2014; van de Werken, et al. 2015). In this case, heterochromatin establishment on paternal satellites is Polycomb-independent. However, Polycomb complexes are known to assemble on human satellites when canonical heterochromatin is perturbed in other contexts. HSATII DNA is frequently demethylated in human cancer cells, and this coincides with the accumulation of PRC1 and the H2Aub mark it deposits (Hall, et al. 2017). Whether PRC1 is recruited through direct recognition of HSATII DNA in this context is unknown, but one study suggests that it might be (Arends, et al. 2026). Conservation of Polycomb packaging of satellite DNA in the absence of canonical heterochromatin raises the possibility that Polycomb substitutes for H3K9me3 at satellites in the early embryos of species other than mouse.

We suggest similar principles likely govern de novo H3K9me3 heterochromatin in early embryos. Evidence for direct sequence recognition comes from *Drosophila*, where the pioneer factor GAGA factor binds the AAGAG satellite and is required to silence repeat-rich genomic regions, where H3K9me3 is established in the embryo (Gaskill, et al. 2023). A growing number of proteins that directly recognize satellite DNA and recruit the H3K9me3 machinery could plausibly play similar roles (Masumoto, et al. 1989; Okada, et al. 2007; Ma, et al. 2024). Because these factors all depend on specific sequence motifs, they may coevolve with satellite DNAs and could drive incompatibilities in hybrids. Those heterochromatin packaging proteins with signatures of adaptive evolution are potential starting points to explore this possibility.

As an alternative to direct satellite DNA sequence recognition by heterochromatin-nucleating proteins, satellite transcripts have been proposed as a plausible route for de novo H3K9me3 deposition by either directly or indirectly recruiting the SUV39 histone methyltransferases (Maison, et al. 2002; Muchardt, et al. 2002; Johnson, et al. 2017; Shirai, et al. 2017; Velazquez Camacho, et al. 2017). Consistent with this possibility, a burst of satellite transcription occurs before heterochromatin is re-established at paternal satellites in mouse embryos (Probst, et al. 2010; Casanova, et al. 2013). Under this model, satellite sequence evolution can potentially change the propensity of satellites to be transcribed via recognition by transcription factors, thereby altering heterochromatin formation that subsequently silences satellite transcription (Bulut-Karslioglu, et al. 2012; Lo, et al. 2026). Alternatively, maternally deposited small RNAs cognate to satellite DNA may seed heterochromatin packaging in the early embryo. In *Drosophila*, maternally deposited Piwi-piRNA complexes silence transposons by base-pairing with their nascent transcripts in embryos and directing H3K9me3 (Fabry, et al. 2021). Because the Rsp and 1.688 satellites are also transcribed and processed into piRNAs in ovaries (Usakin, et al. 2007; Wei, et al. 2021), the same maternally inherited machinery could potentially establish heterochromatin at satellite DNA in *Drosophila* embryos.

Under the coevolutionary framework, the apparent conservation of pericentric heterochromatin masks the recurrent antagonism between satellite DNA and the trans-acting proteins that maintain heterochromatin. We predict that embryonic de novo establishment is where this coevolution would be most apparent because, at this stage, the buffering provided by epigenetic propagation is absent. Indeed, the embryo relies on maternally provisioned factors that are not adapted to divergent satellites from another species, potentially leading to hybrid dysfunction at this early developmental stage. However, there are no studies to date exploring whether changes in the identity or copy number of a particular satellite can spur adaptation of a particular maternally provisioned heterochromatin protein. Pursuing such a functional link would test the coevolutionary model directly.

## The impact of satellite DNA evolution on pericentromere packaging and chromocenter integrity

Heterochromatin-rich genomic regions are not randomly positioned across the nucleus but instead coalesce at the nuclear periphery, around the nucleolus, and at hubs of clustered heterologous pericentromeres, called chromocenters (Bizhanova and Kaufman 2021; Brandle, et al. 2022). This regionalization of heterochromatin into these distinct, typically phase-separated nuclear compartments concentrates heterochromatin proteins that enhance the distinct molecular features of silent chromatin (Strom, et al. 2017). Investigation of chromocenter formation in inter-species hybrids has offered new insight into how the evolution of heterochromatin-enriched satellite DNAs impact this striking spatial organization.

The DNA satellite sequences common to multiple, heterologous chromosomes can promote inter-chromosomal interactions at chromocenters (Pardue and Gall 1970; Fransz, et al. 2002; Jagannathan, et al. 2018, 2019). For example, the *Drosophila melanogaster* AT-hook protein, D1, binds the [AATAT]_n_ satellite found on multiple chromosomes. Depletion of D1 de-clusters chromocenters. One major consequence of this disruption is the translocation of chromosomes to the nuclear periphery, where they can rupture the membrane of the primary nucleus and form lethal micronuclei. This observation indicates that a satellite-binding protein is critical for chromocenter formation and nuclear membrane integrity, two highly conserved features of eukaryotic nuclei. The rapid evolution of satellite DNA predicts the rapid evolution of D1. Consistently, D1 has been shown to evolve under positive selection (Lin, et al. 2024). However, the *D. simulans* version of D1 has only modest phenotypic consequences when swapped into a *D. melanogaster* fly, suggesting that D1 itself may not evolve to preserve this vital structure. Nevertheless, inter-species hybrid dysfunction implicates satellite DNA as a determinant of chromocenter integrity.

Crosses between *D. melanogaster* females and *D. simulans* males result in sterile hybrid daughters and inviable adult sons. The hybrid sons fail to develop their larval imaginal discs (sacs of progenitor cells that give rise to adult structures) and die soon after. In both the developing female germline and larval imaginal discs, chromocenters de-cluster and micronuclei accumulate, phenocopying the depletion of satellite-binding proteins like D1 ((Fig. 2A.III), (Jagannathan, et al. 2019)). Intriguingly, a gene called *lethal hybrid rescue* (*lhr*), which restores viability to the hybrid sons when mutated in the *D. simulans* fathers, also rescues chromocenter integrity. Notably, *lhr* evolves under positive selection (Maheshwari, et al. 2008; Blum, et al. 2017) and the LHR protein localizes to satellite DNA-rich heterochromatin, suggesting that LHR from *D. simulans* may dominantly disrupt chromocenters in the hybrid. Intriguingly, the *D. simulans* LHR localizes robustly to *D. melanogaster* chromocenters (Brideau and Barbash 2011), suggesting that in the hybrid, the *D. simulans* LHR may block D1 from binding its satellite or instead inhibit vital inter-chromosomal interactions mediated by binding between itself and D1. Future research will reveal the molecular details of the interaction between these two genes, and their direct or indirect interactions with species-specific satellite DNAs.

Analogous to D1 and the [AATAT]n satellite, HMGA1 binds and clusters major satellite DNA into chromocenters in *Mus musculus* (Jagannathan, et al. 2018). In addition to these inter-chromosome interactions, HMGA1 also packages major satellite sequences within individual pericentromeres during female meiosis (Dudka et al. 2025a). Acute depletion of HMGA1 caused a striking stretching of pericentric major satellites, disrupting kinetochore organization and attachment to the meiotic spindle. In contrast to the extensive arrays of major satellite in *M. musculus,* its sister species, *M. spretus*, has minimal major satellite and instead mostly minor satellite arrays at pericentromeres. These *M. spretus* pericentromeric minor satellite arrays are not recognized by HMGA1 and do not stretch upon its depletion. Therefore, satellite DNA evolution appears to impact its recognition by the architectural proteins that package it into individual pericentromeres.

The expression level and amino acid sequence of HMGA1 are conserved between the two species, suggesting that HMGA1 is not coevolving with satellite DNA. However, HMGA2, an ancient paralog of HMGA1 that also specifically recognizes and packages major but not minor satellite, is expressed at higher levels in *M. musculus* oocytes than in *M. spretus* oocytes (Dudka, et al. 2026). Furthermore, HMGA2 depletion disrupts pericentromere packaging in *M. musculus* oocytes with high major satellite copy number, but not in *musculus/spretus* hybrid oocytes with lower copy number. Conversely, HMGA2 overexpression leads to chromosome segregation errors in major satellite-poor *spretus* but not hybrid oocytes. Taken together, these results are consistent with HMGA2 and major satellite abundance coevolving to mitigate the costs of either underpackaging major satellite when HMGA2 is limiting or promiscuously packaging other sequences (such as minor satellite) when HMGA2 is in excess.

## The impact of satellite DNA evolution on genome integrity

Satellite DNA poses distinct challenges to genome integrity. First, secondary structure-prone satellite DNA can impede replication fork progression (Boer, et al. 2025). Stalled forks expose single-stranded DNA and create vulnerable structures. If left unresolved, these replication forks can collapse into double-strand breaks or instead are restarted by error-prone mechanisms, leading to mutations, deletions, repeat copy-number changes, rearrangements, or chromosome mis-segregation. Satellite DNA also challenges DNA repair itself: the many identical or near-identical copies make it difficult to select an appropriate repair template, rendering DNA repair vulnerable to ectopic recombination (Chiolo, et al. 2011; Amaral, et al. 2017). Inappropriate recombination can result in repeat copy-number change and chromosome rearrangements.

The challenges of replication through, and repair of, satellite DNA-rich genomic regions are mitigated by various replication and repair factors. For example, the WRN helicase facilitates replication through AT-rich repetitive DNA (Shah, et al. 2010), while the yeast Srs2 is required for efficient replication through (CGG)n repeats (Anand, et al. 2012). Likewise, specific DNA secondary structures can recruit or engage replication and repair factors, including the MRE11 complex and Mus81-Mms4, which cleave, resect, or remodel these intermediates (Ait Saada, et al. 2021; Noto, et al. 2025). Secondary structures also recruit the mismatch repair (MMR) and the nucleotide excision repair (NER) machinery, which resolve damage at and around these structures and can restrict or promote repeat proliferation. Depletion of MMR protein MSH2, for example, restricts CAG repeat expansions (Manley, et al. 1999) and promotes CTG contractions (Savouret, et al. 2003), while depletion of NER proteins promotes or inhibits CAG deletion events (Lin, et al. 2006; Szwarocka, et al. 2007). Interactions between these DNA repair factors and these DNA satellites (and likely others) raise the possibility that rapidly evolving satellite DNA may drive the rapid evolution of DNA repair factors.

Intriguingly, evolution under positive selection is pervasive across diverse DNA repair pathways. These pathways include non-homologous end-joining in *Drosophila* (Anderson, et al. 2009; Lee, et al. 2017), yeast (Sawyer and Malik 2006), and primates (Demogines, et al. 2010; Rowley, et al. 2021) and homologous recombination in *Drosophila* and primates (Lou, et al. 2014; Lee, et al. 2017). DNA damage sensors like ATR and ATM also appear to evolve adaptively (Lee, et al. 2017). Finally, the repair factor, SPARTAN, which proteolyzes covalent crosslinks between DNA and proteins that block replication, evolves adaptively across *Drosophila*.

The most compelling functional evidence to date of satellite DNA-driven DNA repair evolution comes from this “DNA–protein crosslink” repair enzyme Spartan/Maternal Haploid (Delabaere, et al. 2014; Stingele, et al. 2017). DNA–protein crosslinks form as a normal, reversible step of various DNA-templated functions, including DNA replication and transcription. However, enzymatic errors and mutagens can render these transient covalent bonds permanent. Irreversible DNA–protein crosslinks block DNA replication, transcription, and other DNA transactions. Spartan family members, including the proteins Spartan and GCNA, proteolyze the crosslinked protein, enabling the otherwise blocked DNA transactions to proceed. These essential proteins act genome-wide – across both conserved and unconserved DNA sequences – suggesting that they should evolve under purifying selection. New data suggest instead that satellite DNA evolution shapes both the sequence and functional diversity of the Spartan family.

Spartan/Maternal Haploid (MH hereafter) has evolved adaptively between *D. melanogaster* and *D. simulans* (Brand and Levine 2022)*.* Replacing the native *D. melanogaster mh* with the divergent *D. simulans* version leads to profound DNA damage in the female germline, which reduces egg production. Germline genome integrity and egg production can be rescued by deleting the *D. melanogaster*-specific 11 Mb *359 bp* satellite DNA array, consistent with an incompatibility between the *D. melanogaster*-specific satellite and the *D. simulans*-specific repair protein. Intriguingly, these phenotypes can also be reversed by overexpressing Topoisomerase II (Top2), a well-known target of Spartan/MH that transiently crosslinks to DNA to resolve topological stress. Together, these data suggest that the *D. simulans* version of MH over-actively cleaves Top2-*359 bp* crosslinks, impairing Top2-mediated disentanglement of the large *359 bp* satellite array (Fig. 2B.II). Under this model, MH evolved reduced enzymatic activity (dotted outline, Fig. 2B.II) along the *D. melanogaster* lineage, promoting longer Top2 residence time at the *D. melanogaster*-specific *359 bp* satellite array. These data provided the first experimental evidence of satellite DNA-mediated DNA repair evolution and also implicated a second protein in the evolution of this pathway: Top2.

Top2, like MH, evolves adaptively (Brand and Levine 2022)*.* Unlike MH, expressing the *D. simulans* Top2 in *D. melanogaster* ovaries has no detectable impact on female germline genome integrity; however, maternal deposition of this heterologous protein causes embryonic lethality in *D. melanogaster* (Brand, et al. 2026). The source of this Top2-mediated embryonic lethality is *359 bp*. In the presence of the *D. simulans* Top2, the *D. melanogaster 359 bp* becomes trapped as a chromatin bridge (Fig. 2B.III). Deleting the *359 bp* array restores faithful chromosome segregation and embryonic viability, consistent with a lethal inter-species genetic incompatibility between *D. simulans* Top2 and *D. melanogaster-*specific *359 bp*. The data further suggested that the heterologous *D. simulans* Top2 fails to efficiently relieve topological challenges at this unfamiliar satellite array.

The inability of *D. simulans* Top2 to process *359 bp* is reminiscent of the F_1_ hybrid embryonic lethality that arises from crossing pure *D. simulans* mothers and pure *D. melanogaster* fathers (Fig. 2A.IV, (Ferree and Barbash 2009)), a reproductive barrier documented over 100 years ago by Alfred Sturtevant (Sturtevant 1920). Excitingly, maternal deposition of transgenic *D. melanogaster* Top2 by *D. simulans* mothers into hybrid embryos partially rescues hybrid viability, consistent with Top2 contributing to a long-studied case of reproductive isolation (Brand, et al. 2026). These data further support the notion that species-specific satellite DNAs require species-specific alleles of genes that maintain genome integrity.

How generalizable is DNA repeat-mediated adaptive evolution of DNA–protein crosslinking and repair? The DNA sequence footprint of rapid adaptation of the Spartan/MH-Top2 pathway appears to transcend the *D. melanogaster-D. simulans* species pair. The Spartan family evolves rapidly across multiple distinct clades from the *Drosophila* phylogeny that spans 60 million years of evolution (Brand, et al. 2024). The nature of this rapid evolution includes the accumulation of amino acid-changing mutations in multiple family members across multiple clades, and also striking gene duplication, divergence, and deletion dynamics. These genes also undergo expression evolution – daughter genes recurrently evolve testis-specific gene expression from parent genes expressed in the ovary. Moreover, the gene encoding the Spartan family member Germ cell nuclear acidic peptidase, or Gcna, has duplicated extensively in *D. obscura*, with at least 46 copies of its SprT-like domain occurring along the X-chromosome (Katoh, et al. 2025). These duplicates likely act as selfish elements that underlie the well-documented, non-Mendelian X transmission. The authors speculated that the aberrantly high dose of these Spartan family genes over-actively cleave Top2 crosslinks along the satellite DNA-rich Y chromosome, which would lead to persistent topological challenges.

A final piece of evidence that DNA–protein crosslink repair pathway evolution transcends *Drosophila* comes from the substrate of Spartan/MH, Top2. The unexpected discovery of Top2 adaptive evolution along the *D. simulans* lineage is also apparent across rodents and primates (Brand, et al. 2026). Intriguingly, Top2 is enriched at mouse pericentromeres, raising the possibility that this repeat-rich genomic region may drive Top2 innovation even in mammals (see condensin section). Future work will establish the extent to which DNA repeat evolution has shaped DNA–protein crosslink repair across eukaryotes. Regardless, these studies suggest that it is not uncommon for a broadly distributed, globally acting DNA repair factor to evolve rapidly under pressure from a specific locus such as a satellite DNA array.

## Emerging themes from, and future challenges for, studying the functional significance of satellite DNA evolution

Recent investigations of the functional consequences of satellite DNA evolution offer robust support for a long-standing conceptual model of antagonistic coevolution. Indeed, this model predicts that inter-species gene swaps and hybrids would lead to incompatibilities that compromise chromosome stability and genome integrity. However, these mechanistic studies offer several important elaborations of this model. For example, the classic molecular interface of intra-genomic coevolution lies between DNA and protein. Specifically, a DNA-binding protein recurrently innovates to recognize the sequence of ever-evolving DNA repeats (Henikoff, et al. 2001; Jacobs, et al. 2014). However, current evidence implicates additional agents of selection, beyond the primary DNA sequence, including DNA shape (e.g., minor groove width) and the secondary structures (hairpins, G4s Dudka et al. 2025a, Thakur et al. 2021)) that emerge from primary satellite DNA sequence. Moreover, proteins that appear to coevolve with satellite DNA, for example Spartan/Maternal Haploid and Cenp-T (see above), do not encode sequence-specific DNA-binding domains or even DNA-binding domains at all. This observation further implicates satellite sequence evolution as an indirect force shaping centromere proteins, pericentromere proteins, and other factors that engage repeat-rich genomic regions. Finally, studies in hybrids suggest that the up- or down-regulation of these heterochromatin-interacting proteins, in addition to rapidly changing residues – DNA binding or otherwise – may also be responsive to satellite DNA evolution (e.g., mouse condensins, see above).

Another feature of long-standing conceptual models of antagonistic coevolution is specialization of the host protein on a DNA satellite or satellite-rich genomic region. Indeed, how a broadly acting protein could be sufficiently unconstrained to evolve in response to individual, rapidly evolving satellites or satellite arrays is not obvious. However, the studies reviewed here suggest that even proteins that perform essential functions across the genome, at both euchromatin and heterochromatin alike, are subject to adaptive evolution under pressure from specific satellite DNAs. One resolution to this paradox is that heterochromatin functions require specialization at repetitive DNA, driving protein innovation, while euchromatin is permissive to these changes so that a broadly conserved function required across the unique DNA is maintained.

The idea of specialization versus permissiveness is also reflected in the developmental specificity of positively selected functions of the repeat-interacting proteins. The early embryo has emerged as a uniquely sensitive developmental stage when satellite DNA can perturb essential functions, suggesting that the strict requirements of the early embryo have an outsized effect on a protein’s sequence or its regulation. The de novo establishment of the epigenomic landscape, the radical chromatin remodeling, and the uniquely rapid cell cycles of the early embryo may all contribute to this developmental “pressure point.” Later stages of development effectively tolerate the tweaks made by evolution under pressure from satellite DNA.

Although it is increasingly clear that satellite DNA evolution impacts fundamental biology, the forces that led to the rapid evolution of satellite DNAs themselves are often disconnected from these functional tests. Both neutral and non-neutral processes may lead to the fixation of a satellite array. The most commonly cited non-neutral force is the increase in frequency of selfish satellite DNAs that achieve non-Mendelian transmission. Many studies of host protein adaptation to novel DNA satellites presume this evolutionary history, but explicit tests are still lacking. This remains a major challenge. The disparate timescales of selfish element dynamics (short) and adaptive protein evolution probed in inter-species studies (comparatively long) must be overcome to establish these links.

Looking forward, the study of satellite DNA evolution is only accelerating. The recent deluge of telomere-to-telomere assemblies is rapidly revealing the complement of repetitive DNA sequences that may (or may not) impact vital functions. Continued use of inter-species hybrids and gene swaps is likely to reveal even more biological pathways subject to innovation under pressure from satellite DNA. Excitingly, we are also entering a new era of DNA repeat manipulation, where CRISPR/Cas9-mediated allelic series of varying satellite copy numbers offer us, for the first time, rigorous assessments of the functional significance of repetitive DNA and with that, its evolution (Eickbush, et al. 2026; Gilmour, et al. 2026; Wang, et al. 2026). The discovery of DNA repeat-associated proteins is also rapidly accelerating with advances in proteomic analysis of chromatin packaging of repetitive DNA (Becker, et al. 2017; Chavan, et al. 2025). Subjecting these proteins to population genetic and molecular evolution analyses will offer new candidate host factors that are antagonized by satellite DNA. These host factors will reveal new biology shaped by this antagonistic evolution.

## Data Availability

No datasets were generated or analysed during the current study.
